# Supplementary material for: Unique Nucleotide Polymorphism of African Swine Fever Virus Circulating in East Asia and Central Russia
Source: Viruses. 2024 Dec 11;16(12):1907. doi: 10.3390/v16121907 (PMC11680119; doi:10.3390/v16121907)
Supplement: Supplementary file 1 [file viruses-16-01907-s001.zip › Supplementary Table S1.pdf]

Supplementary Table S1. Characteristics of isolates used in the study

Isolate name	Host	Sample collection date	Place of collection	Viral load in PBMC (lg HAD_{50}/ml ± SD)	Accession number (Genbank)
ASFV/Amur_2021/WB-10591	wild boar	18.08.2021	Amur region, Bureysky district, forest area	6,89±0,31	PP982225.1
ASFV/Amur_2021/WB-10595	wild boar	28.09.2021	Amur region, Shimanovsky district, village Novovoskresenovka	7,12±0,26	PP982226.1
ASFV/Amur_2022/WB-905	wild boar	05.10.2021	Amur region, Arkharinsky district, mount Moscow, preserve " Andreevsky "	7,08±0,11	PP982227.1
ASFV/Amur_2022/WB-909	wild boar	05.10.2021	Amur region, Zavitinsky district, Verkhne-Zavitinsky preserve	6,34±0,14	PP982228.1
ASFV/Amur_2022/WB-911	wild boar	05.10.2021	Amur region, Shimanovsky district, village Ushakovo	6,76±0,31	PP982229.1
ASFV/Belgorodskaya_2021/DP-11838	domestic pig	22.09.2021	Belgorod region, Korochansky district, pig industry farm	6,47±0,22	PP982230.1
ASFV/Belgorodskaya_2021/DP-11869	domestic pig	25.09.2021	Belgorod region, Gubkinsky district, town Gubkin, meat processing plant (The meat is believed to have come from the Novgorod region, Novgorod district, Chechulino village)	6,87±0,15	PP982231.1
ASFV/Bryanskaya_2021/DP-18	domestic pig	30.08.2021	Bryansk region, Zhukovsky district, village Letoshniki, pig industry farm	8,32±0,43	PP982232.1
ASFV/Bryanskaya_2021/DP-8823	domestic pig	02.09.2021	Bryansk region, Zhukovsky district, village Letoshniki, private farm	7,64±0,28	PP982233.1
ASFV/JAO_2020/DP-6768	domestic pig	18.08.2020	Jewish Autonomous Region, Smidovichi district, village Belgorodskoe, private farm	6,22±0,45	PP982234.1
ASFV/Khabarovsk_2020/DP-11562	domestic pig	22.10.2020	Khabarovsk region, Vyazemsky district, village Zabaikalskoye, private farm	7,65±0,35	PP982235.1
ASFV/Khabarovsk_2020/WB-11558	wild boar	12.11.2020	Khabarovsk region, Khabarovsk district, village Kazakevichevo, forest area	7,18±0,09	PP982236.1
ASFV/Khabarovsk_2021/WB-3967	wild boar	27.03.2021	Khabarovsk region, Khabarovsk district, preserve Bolshekhtsirsky	6,98±0,17	PP982237.1
ASFV/Khabarovsk_2022/DP-1658	domestic pig	04.02.2022	Khabarovsk region, Name of Lazo district, village Мухен, private farm	8,08±0,38	PP982238.1
ASFV/Khabarovsk_2022/WB-1650	wild boar	25.03.2022	Khabarovsk region, Khabarovsk district, forest area, near the upper Kukan River	7,45±0,38	PP982239.1
ASFV/Leningrad_2019/WB-789	wild boar	24.02.2019	Leningrad region, Luzhsky district, preserve «Mshinskoye bog»	7,32±0,11	PP982240.1
ASFV/Permskyi_2021/DP-9916	domestic pig	27.09.2021	Perm region, Krasnokamsky district, village Karabai, private farm	8,08±0,19	PP982241.1
ASFV/Primorsky_2021/DP-9778	domestic pig	23.07.2021	Primorsky region, Kavalerovsky district, village Kavalerovo, private farm	6,55±0,09	PP982242.1
ASFV/Primorsky_2021/WB-9786	wild boar	05.09.2021	Primorsky region, Shkotovsky district, village Shkotovo, forest area	6,73±0,32	PP982243.1
ASFV/Pskovskaya_2021/DP-9008	domestic pig	11.09.2021	Pskov region, Ostrovsky district, village Malya Guba, pig industry farm	8,70±0,16	PP982244.1
ASFV/Sverdlovskaya_2021/DP-9914	domestic pig	26.09.2021	Sverdlovsk region, Kamyshlovsky district, village Kalinovskoe, private farm	6,92±0,10	PP982245.1
