# Supplementary material for: Unique Nucleotide Polymorphism of African Swine Fever Virus Circulating in East Asia and Central Russia
Source: Viruses. 2024 Dec 11;16(12):1907. doi: 10.3390/v16121907 (PMC11680119; doi:10.3390/v16121907)
Supplement: Supplementary file 1 [file viruses-16-01907-s001.zip › Supplementary Table S2.pdf]

Supplementary Table S2. Characterization of isolates imported from GenBank

№	Isolate	Accession number (Genbank)
1	Georgia 2007/1	NC_044959.2
2	ASFV/Primorsky_2019/WB-8235	OP510032.1
3	ASFV/Zabaykali 2020/WB-5314	MZ325862.1
4	ASFV/Zabaykaly_2020/DP-4905	OP510033.1
5	ASFV/Kaliningrad 17/WB-13869	OM799941.1
6	ASFV/Kaliningrad 18/WB-9734	OM966721.1
7	ASFV/Kaliningrad 18/WB-9763	OM966717.1
8	ASFV/Kaliningrad 18/WB-9766	OM966718.1
9	ASFV/Kaliningrad 18/WB-12523	OM966714.1
10	ASFV/Kaliningrad 18/WB-12516	OM966720.1
11	ASFV/Kaliningrad 18/WB-12524	OM966715.1
12	ASFV/Kaliningrad 18/WB-9735	OM966716.1
13	ASFV/Ulyanovsk 19/WB-5699	MW306192.1
14	ASFV/Kabardino-Balkaria 19/WB-964	MT459800.1
15	ASFV/Odintsovo 2014/WB	KP843857.1
16	ASFV POL/2015/Podlaskie	MH681419.1
17	ASFV Pol16_20538_o9	MG939584.1
18	ASFV Pol16_29413_o23	MG939586.1
19	ASFV Pol16_20540_o10	MG939585.1
20	ASFV Pol16_20186_o7	MG939583.1
21	ASFV Pol17_31177_O81	MT847622.1
22	ASFV Pol17_04461_C210	MG939588.1
23	ASFV Pol17_03029_C201	MG939587.1
24	ASFV Pol17_05838_C220	MG939589.1
25	ASFV Pol18_28298_O111	MT847621.1
26	ASFV Pol19_53050_C1959/19	MT847623.1
27	ASFV Pol17_55892_C754	MT847620.1
28	ASFV China DB/LN/2018	MK333181.1
29	ASFV China Pig/HLJ/2018	MK333180.1
30	ASFV/LT14/1490	MK628478.1
31	ASFV CzechRepublic 2017/1	LR722600.1
32	ASFV Moldova 2017/1	LR722599.1
33	ASFV Belgium 2018/1	LR536725.1
34	ASFV Germany 2020/1	LR899193.1
35	ASFV/Kaliningrad 19/WB-10168	OM966719.1
